# The transcultural adaptation and validation of the Chinese version of the Oral Health Literacy Scale for Diabetic Patients

**DOI:** 10.1186/s12903-024-03965-9

**Published:** 2024-02-07

**Authors:** Ying Zhao, Hang Zhao, Hongyu Yu

**Affiliations:** 1 Department of Nursing, Jinzhou Medical University, Jinzhou, China; 2 Department of Rehabilitation Medicine, General Hospital of Northern Theater Command, Shenyang, China

**Keywords:** Reliability, Validity, Psychometric validation, Cross-cultural adaptation, Factor analysis, Oral health literacy

## Abstract

**Background:**

Diabetic patients’ oral health concerns are a reality for every diabetic patient in China. The attitudes of diabetic patients toward early identification of oral literacy influence oral literacy in diabetes patients. Diabetes patients' oral health literacy is critical for providing focused education and therapies. However, no instrument exists to measure oral health literacy in Chinese diabetic patients. In this study, the English version of the oral health literacy among people living with diabetes (OHLD) scale was cross-culturally validated to provide a reliable tool for assessing the oral health literacy of diabetic patients in China.

**Objective:**

The oral literacy among people living with diabetes (OHLD) scale was Chineseized and its reliability and validity tested, and the OHLD scale was revised to test the reliability and validity of the Chinese version of the OHLD scale and to provide a tool for assessing the oral health literacy of diabetic patients in China.

**Methods:**

A modified version of the Brislin translation model was used, cross-cultural adaptation was performed through expert consultation and pre-survey, and expert opinion was used to assess content validity to form the Chinese version of the Oral Health Literacy Scale for Diabetic Patients, which was administered to 420 diabetic patients in two tertiary hospitals in Liaoning Province from March to August 2023. The reliability of the scale was tested. SPSS 25.0 and AMOS 23.0 were used to analyze the data.

**Results:**

The Chinese version of the OHLD scale consisted of three dimensions and 10 entries. Structure of the validity analysis: three factors were extracted from the exploratory factors with a cumulative variance contribution of 79.794%; Content validity results:An item’s content validity index (I-CVI) was 0.857 to 1 at the entry level, and the content validity index of the scale (S-CVI) was 0.928 at the scale level. The results of the reliability analyses were: the Cronbach's alpha coefficient for the total scale was 0.908; the Cronbach's alpha coefficients for the factors and dimensions were 0.853 to 0.922; the split-half reliability was 0.827; and the test–retest reliability was 0.848. The results of the validation factor analysis showed that (χ^2^/df) was 1.430, the root mean square of the error of approximation (RMSEA) was 0.045, the comparative fit index (CFI) was 0.989, and the Tucker Lewis index (TLI) was 0.985, which showed that the model had an overall good fit.

**Conclusion:**

The Chinese version of the OHLD scale has good reliability and validity and can be used as a valid tool for assessing diabetes mellitus patients in China.

## Introduction

Due to aging and lifestyle changes, the incidence of various chronic diseases is increasing year by year. These include diabetes, chronic obstructive pulmonary disease (COPD), and cardiovascular disease [1]. Diabetics have been reported to develop a variety of symptoms, including cardiovascular symptoms [2], gastrointestinal [3], and urinary symptoms [4]. The complexity of the etiology of diabetes and the diversity of symptoms deserve our consideration. Diabetic stigma has attracted the attention of scholars [5]. With the aging of populations worldwide, the incidence and prevalence of chronic diseases and the economic burden on healthcare providers remain very challenging [6]. In China, diabetes has become a very common disease, and in the wake of the COVID-19, the degree of prognosis for people with diabetes is not very favorable. In Ghana, for example, patients' and health care providers' self-management of diabetes was studied, which informs diabetes care in other middle-income countries [7]. Improving the health of the population is the main objective of the health system [8]. Diabetes has become a major chronic disease affecting people's lives because of its high cost [9]. According to the World Health Organization, health literacy is the ability of people to acquire knowledge, understand it, and apply it. Health literacy includes personal health status, culture, and experience, and nurses use this information to provide appropriate care and improve people's health [10]. According to Lauren, health literacy is a skill that includes the ability to read, write, compute, and communicate [11]. Health literacy is a complex topic that requires health care providers to conduct health promotion [12]. In developed countries, health literacy has been prioritized, both in policy, practice, and research [13]. Lower levels of health literacy have been reported among older persons, minorities, and persons of lower socioeconomic status. Personalized health care can promote positive health outcomes and contribute to more effective diabetes management [14]. Diabetes self-care is associated with health literacy [15]. Research has shown that health literacy is one of the major influences on chronic disease. It has been shown that increasing the level of health literacy in at-risk populations is feasible for slowing the progression of chronic diseases [16]. In the context of a healthy China, health knowledge is being promoted to raise awareness of health care for people with diabetes. Oral problems can affect the mood of diabetic patients and undermine their quality of life, so it is important that the diabetic population has the ability to recognize the need for oral problems and receive the necessary care. Therefore, appropriate oral hygiene education needs to be provided to diabetic patients to increase their awareness of self-protection. Oral health education can be integrated into chronic disease management [17]. Oral health literacy has been shown to reduce oral health disparities and promote oral health [18]. Oral health literacy is related to an individual's oral health management, doctor-patient communication, behaviors and attitudes towards oral health, and the health care system [19]. With oral health literacy, it is important for individuals to not only understand the essentials but also adopt behaviors that carry out oral health [20]. For example, Ayesha claims that although many people claim to value oral health, they don't care about oral problems until they arise [17]. It has been shown that the burden of oral disease is particularly severe in developing countries [21]. Diabetes can cause immune and salivary dysfunction, which in turn increases oral diseases such as dental caries and periodontitis, which in turn affect oral health [22]. Periodontal disease is a recognized complication of diabetes [23, 24]. Diabetes can affect oral health, and there is a bidirectional relationship between oral health and glycemic control [25]. General health literacy measures may not adequately reflect situation-specific health literacy skills. Therefore, it is important to measure situation-specific health literacy so that caregivers can provide optimal care. The OHLD scale is based on the theoretical model proposed by Sørensen et al. [26]. The evaluation of health literacy consists of four dimensions: access to health-related information, understanding, evaluation and application. In line with this theory, in order to assess the oral health literacy of diabetic patients, Brazilian scholar Dr. Andrea Maria et al. developed the OHLD scale. However, oral health literacy remains unexplored in Chinese diabetic patients. There is no validated psychometric tool to estimate oral health literacy among Chinese diabetic patients. The aim of this study was to translate and cross-culturally debug the scale, introduce the English version of the OHLD scale into China, and assess the reliability and validity of the translated scale among Chinese diabetic patients.

## Materials and methods

### Design and participants

The aim of this study was to translate the English version of the OHLD scale and test the reliability of its Chinese version. The study was conducted from March to August 2023 in two tertiary care hospitals in Liaoning Province. Participants included were (1) over 18 years old, (2) diagnosed with diabetes mellitus by a physician, and (3) Voluntary participation in the study. Exclusion criteria: (1) severe visual/auditory impairment; (2) mental illness and cognitive impairment; (3) questionnaire completion less than 100% removed. According to the rules of the factor analysis procedure, a minimum of three respondents per item is required (Kline, 1998), but a larger sample size is desirable. In this study, 10 respondents were required for each item to ensure accuracy [27]. Finally, 420 diabetic patients were selected to participate in the questionnaire. We collected demographic and disease-related information about the participants.

### Translation, countertranslation, and cross-cultural adaptation of the Oral Health Literacy Scale for diabetic patients

Translation and adaptation of the English version of the OHLD scale have been carried out with the permission of Dr. Andréa Maria [28]. The OHLD scale has been translated using the Brislin double inverse translation method. First, a Chinese professor majoring in English and a graduate student majoring in English who had passed the sixth grade of English and had experience studying abroad translated the OHLD scale into Chinese. Then, two native English-speaking foreign teachers did the reverse translation. Moreover, And two experts were also invited to culturally debug the translated scale. Ten diabetic patients were recruited to pre-survey the draft translation [29], and their opinions and suggestions were fully listened to, which finally resulted in the Chinese version of the OHLD scale. In order to adapt to the Chinese cultural background, some modifications were made to the entries in the scale, called cultural adaptation. (1) Expert consultation:Seven experts were invited to review the questionnaire and judge the appropriateness of each topic. The seven experts included two endocrinologists, one psychologist, two clinical care managers, one professor of English, and one specialist nurse, all with postgraduate degrees or above. These criteria were used to choose the experts: (1) extensive expertise in diabetes, oral problems, and nursing care; (2) familiarity and experience with the steps and processes of scale translation; (3) postgraduate education and more than 10 years of experience; (4) voluntary participation in this study. The final version was created by adapting various elements to Chinese culture and language habits. (2) Pre-survey: Ten diabetic patients were conveniently selected to answer the preliminary questionnaire to find out their understanding of the scale. The subjects were first explained, and informed consent was obtained. The opinions and suggestions of the subjects were fully heard. Finally, the Chinese version of the OHLD scale was formed.

### Questionnaire design

Our questionnaire on general demographic characteristics and disease-related information was developed after extensive reading of the literature. The general questionnaire contained five variables: age, gender, mode of payment for health care [30], education, and occupation. Disease-related information included the number of years since the diagnosis of diabetes mellitus, genetic history, number of real teeth [31], and participation in oral health literacy activities.

### The OHLD scale

Dr. Andréa Maria and colleagues developed the 10-item OHLD scale [32]. A scale is used to comprehensively assess oral health literacy in diabetic patients. The OHLD scale consists of three dimensions: (1) information acquisition (3 items), (2) information comprehension (5 items), and (3) information application (2 items). A five-point Likert scale was used (1 = strongly disagree, 2 = disagree, 3 = neutral, 4 = agree, 5 = strongly agree). The higher the score, the more diabetic patients knew about oral health literacy. The raw scales had acceptable internal consistency, with a Cronbach's alpha of 0.908 for the overall scale.

### Data collection

All researchers underwent uniform professional training and recruited participants from two tertiary hospitals in two cities in Liaoning Province. Before collecting the data, the researcher explained the purpose of this survey to the participants and ensured that the collected data would not be disclosed. The data was used only for this investigation. After the subjects gave their informed consent, a paper version of the questionnaire was distributed and completed. After conducting rigorous screening, a total of 420 valid questionnaires were finally collected. The collected data were collated and numbered, and two-person data entry was used to ensure the accuracy and completeness of the data. No information would be disclosed without the consent of the respondents. In order to assess the re-test reliability, two weeks later, 40 survey respondents were randomly selected from the current survey respondents to answer the Chinese version of the OHLD questionnaire at the same location to assess the reliability of the questionnaire.

### Statistical analysis

SPSS 25.0 and AMOS 23.0 were used to analyze the data in this study. Regarding the general demographic characteristics of diabetic patients, frequency and composition ratios were used to describe them. We considered *p* < 0.05 to be significant. Item analysis was used to assess the quality of the items, and expert correspondence was used to assess the appropriateness of each topic. Exploratory factor analysis was used to explore the underlying factor structure of this scale. Validated factor analysis was conducted using AMOS 23.0 to explore the structural validity of this scale. This scale's reliability was assessed using retest reliability and internal consistency analysis.

### Item analysis

Item analyses were designed to determine the differentiation and relevance of the scales. The appropriateness of the scale entries was tested using the method of correlation coefficients between the entries and the total score and the critical ratio method. The Critical Ratios(CR) method is to find the decision value of each item in the questionnaire and sort it according to the total score. Before and after, 27% is picked as the high and low groups, and the scores of each question in the two groups are compared using a T-test [33]. Deletion of entries that did not reach the decision value [34]. At the same time, we also use the total correlation method, that is, the correlation coefficient between each item and the total score, to determine the homogeneity of the item. Item-total correlation coefficients ≥ 0.4 were considered appropriate, and entries that did not reach significance were deleted. The Cronbach's α coefficient was calculated if an item was removed. If the Cronbach's coefficient of an entry increased significantly after deletion, it was recommended that the entry be deleted, which means that the internal correlation of the entry decreased and should be deleted. This was done to determine whether the item could be retained on the translated scale.

### Reliability analysis

The reliability test of this scale was evaluated using Cronbach's coefficient, split-half reliability, and retest reliability. Cronbach's α coefficient, split-half reliability coefficient, and retest reliability coefficient should be greater than 0.7 [35]. The Chinese version of the OHLD scale was divided into two parts according to the odd and even number of entries to calculate the split-half reliability [36]. After two weeks, 40 diabetic patients who had completed this scale were randomly selected to calculate the retest reliability.

### Validity analysis

In this study, we used a four-point Likert scale (1 = irrelevant, 2 = weakly relevant, 3 = strongly relevant, and 4 = highly relevant) to collect experts' responses. Irrelevance and weak relevance were scored as 0, and strong relevance and high relevance were scored as 1. The content validity of this scale was evaluated by calculating the item content validity index (I-CVI) and scale content validity index (S-CVI) [37]. I-CVI denotes the content validity index of each entry, calculated as the percentage of the number of all experts by collecting strongly and highly relevant expert ratings. S-CVI denotes the average CVI value of each entry, calculated as the average of the I-CVI of each entry in the scale. In assessing the underlying factor structure of the scale, We randomly assigned 420 cases to two equal groups, part of which we did exploratory factor analysis (EFA), using principal component analysis with maximum variance orthogonal rotation [38]. In general, contributions in excess of 50 percent are considered acceptable, and contributions in excess of 70 percent are considered appropriate [33, 39]. Validated factor analysis of the Chinese version of the OHLD scale was performed using AMOS. The chi-square degrees of freedom ratio (χ^2^/DF < 3), goodness-of-fit index (GFI), adjusted goodness-of-fit index (AGFI), incremental fit index (IFI), Tucker Lewis index (TLI), and comparative fit index (CFI) should all be greater than 0.9 [33, 40]. The root mean square error of approximation (RMSEA) should be less than 0.08, which indicates a good fit [41].

## Results

### Descriptive statistics

This study comprised 420 diabetes patients in total, of whom 188 (44.8%) were male and 232 (55.2%) were female. Participants older than 65 accounted for 26.9%. 28.6% of the participants had an education beyond junior high school. The largest proportion of participants were farmers (16.9%); and 66.3% had not participated in oral health education activities. This study meets the standards of the Helsinki Declaration. Table 1 displays specific sociodemographic statistics.

**Table 1:** Frequency distribution of demographic characteristics (*n* = 420)

Factors	Group	*n*	%
Age	< 35	109	26
	35–50	101	24
	50–65	97	23.1
	> 65	113	26.9
Gender	Male	188	44.8
	Female	232	55.2
Payment of medical expenses	worker with medical insurance	181	43.1
	medical insurance for residents	55	13.1
	new rural cooperative medical system	79	18.8
	self-paying	68	16.2
	else	37	8.8
Education level	Primary school and below	54	12.9
	junior high school	120	28.6
	High school or technical secondary school	81	19.3
	Junior college education	57	13.6
	Undergraduate education	92	21.9
	Postgraduate education and above	16	3.8
Occupation	worker	62	14.8
	farmer	71	16.9
	student	21	5
	Employees of enterprises and public institutions	62	14.8
	Business or service workers	19	4.5
	professional and technical staff	45	10.7
	the emeritus and retired	58	13.8
	freelancer	34	8.1
	else	48	11.4
Diagnosis of diabetes for how many years	< 5 year	348	82.9
	5–10 year	72	17.1
Family genetic history	yes	119	28.3
	no	301	71.7
Number of real teeth available	none	46	11
	1–9	49	11.7
	10–19	90	21.4
	20–32	235	56
Oral health education activity	yes	154	36.7
	no	266	63.3

### Item analysis

The Chinese version of the OHLD scale has 10 items with Critical Ratios (CR) ranging from 12.452 to 30.088. CR values greater than 3 indicate that the items are strongly discriminatory. The correlation coefficients (r) between the entries and the total score ranged from 0.633 to 0.822 and were all greater than 0.2, indicating a direct correlation between the questions and the scale. After deleting the entrie did not exceed the Cronbach's α value for the scale (0.908; Table 2).

**Table 2:** Item analysis for the Chinese version of the OHLD scale

Item	Critial ratio	The correlation coefficient between the Item and the total score	Cronbach’s alpha, if an item deleted
1	16.218	0.661	0.904
2	15.471	0.698	0.901
3	18.704	0.731	0.900
4	20.772	0.772	0.896
5	30.046	0.822	0.892
6	27.301	0.818	0.892
7	23.277	0.797	0.894
8	24.939	0.800	0.894
9	12.460	0.633	0.906
10	12.855	0.667	0.904

### Reliability analysis

The Cronbach's α of the Chinese version of the OHLD scale was 0.908, with a dimensional range of 0.853—0.922; the split-half reliability was 0.827; and the retest reliability was 0.848. Table 3 displays specific statistics.

**Table 3:** Reliability analysis for the Chinese version of the OHLD scale

The scale and its dimension	Cronbach’s α	Split-half reliability	Test–retest reliability
The OHLD scale	0.908	0.827	0.848
Factor 1	0.853		
Factor 2	0.922		
Factor 3	0.854		

## Validity analysis

### Content validity analysis

The correspondence of the Chinese version of the OHLD scale by seven experts showed that the I-CVI was 0.857—1.000 and the S-CVI was 0.928. All greater than 0.8 [42]. The results indicated that the Chinese version of the OHLD Scale had sufficient content validity. Table 4 displays specific statistics.

**Table 4:** Content validity analysis for the Chinese version of the OHLD scale

Experts (score)								
Item	1	2	3	4	5	6	7	I-CVI
a1	1	1	1	1	1	1	1	1
a2	1	1	1	1	1	1	1	1
a3	1	1	0	1	1	1	1	0.857
a4	1	1	1	1	1	1	1	1
a5	1	1	1	0	1	1	1	0.857
a6	1	1	1	1	1	1	1	1
a7	0	1	1	1	1	1	1	0.857
a8	1	1	1	1	0	1	1	0.857
a9	1	1	1	1	1	1	1	1
a10	1	1	1	1	1	1	0	0.857

### Exploratory factor analysis

**Fig. 1:**
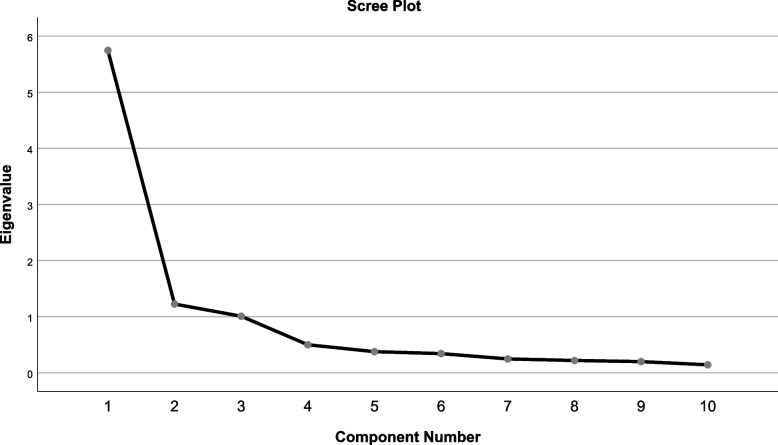
Screen plot of exploratory factor analysis for Chinese version of the OHLD

The Chinese version of the OHLD scale had a KMO value of 0.880 and a Chisquare value of 1492.427 (*p* < 0.001) for the Bartlett's test of sphericity, making it well suited for factor analysis. Three factors with eigenroots > 1 were extracted using variance-maximizing orthogonal rotation (Table 5), and the gravel plot in Fig. 1 explained 79.794% of the total variance.

**Table 5:** Factor loadings of exploratory factor analysis for the Chinese version of the OHLD scale

Item	Factor 1	Factor 2	Factor 3
a1	0.796		
a2	0.87		
a3	0.799		
a4		0.711	
a5		0.8	
a6		0.84	
a7		0.878	
a8		0.832	
a9			0.897
a10			0.853

### Validation factor analysis

**Fig. 2:**
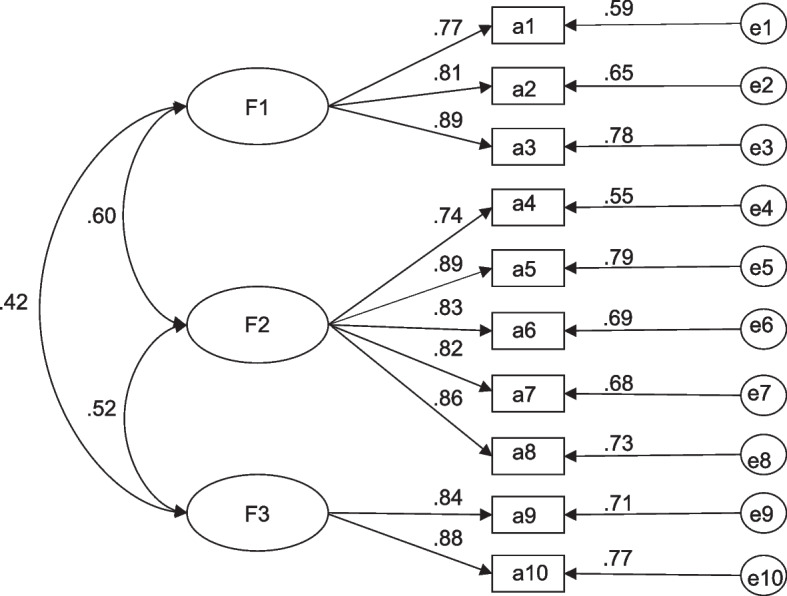
Standardized three-factor structural model of the OHLD (*n* = 420)

The results showed that the CFA-based model fitted the data better (χ^2^ /DF = 1.430 < 5, GFI = 0.960 > 0.9, AGFI = 0.931 > 0.9, CFI = 0.989 > 0.9, RMSEA = 0.045 < 0.08, LI = 0.985 > 0.9). As shown in Fig. 2.

## Discussion

To assess oral health literacy in diabetic patients, the OHLD scale was used cross-culturally and validated with 420 diabetic patients. The Oral Health Literacy Scale for Diabetic Patients was first applied to a Chinese population and had good construct validity and reliability. It can be used to assess the oral health literacy of diabetic patients, which is essential for nurses to conduct health education and improve service quality. We followed the Brislin translation principle [28, 43]. Translation of the English version of the OHLD scale into Chinese Seven experts were invited to assess the semantics, language conventions, professional nature of the OHLD scale, and its content validity. After review by the experts, the items were considered appropriate, and the results showed good content validity. A pre-survey of 40 diabetic patients was conducted, and no ambiguous entries appeared, indicating that the Chinese version of the OHLD scale was easy to understand and reasonably structured. The CR values of the Chinese version of the OHLD scale were all greater than three, proving that the analysis was statistically significant. After deleting each entry, none of the Cronbach's α values exceeded the value of the total scale. In Table 2, some of Cronbach's alpha is higher but has a lower correlation (e.g., item 1), while some of the items have a higher correlation (item 5) but have a lower correlation than item 1. Because the correlation itself is not high, the Cronbach coefficient becomes good if the entry is removed, suggesting that the scale may be likely to perform better if the entry is not in the scale. The correlation itself is high, and if the entry is removed, the Cronbach coefficient becomes bad, indicating that the presence of the entry in the scale is valuable. However, since their correlations are all greater than 0.5 and the Cronbach coefficients are all greater than 0.8, the entry is not considered for deletion. In summary, there is no need to modify the number of item entries in the original scale, and it can be retained in the Chinese version. We also conducted reliability analyses on the Chinese version of the OHLD scale in order to reflect the authenticity of the scale [44]. Internal consistency, retest reliability, and split-half reliability were used to assess the reliability of the Chinese version of the OHLD scale. Internal consistency was expressed as Cronbach's α value, which reflects the homogeneity among the scale items [45]. The Cronbach's alpha for the translation scale in this study was 0.908, and the coefficients for the dimensions ranged from 0.853 to 0.922. Retest reliability in the study refers to the consistency of the results obtained by repeated measurements with the same subjects [46]. In this study, the retest reliability of the Chinese version of the OHLD scale was better than the standardized value, indicating that the scale reliably measured the oral health literacy of diabetic patients, and overall, the Chinese version of the OHLD scale showed good reliability among diabetic patients. Validity refers to the extent to which a measuring instrument can accurately measure the thing being measured [47]. In this study, we evaluated the validity of the Chinese version of the OHLD in terms of content validity analysis and structural validity analysis. Regarding the validity analysis of the Chinese version of the OHLD, seven experts were invited to evaluate the content validity of the Chinese version of the OHLD scale. The study showed that the I-CVI ranged from 0.857 to 1.000; the mean value of the S-CVI was 0.928. All of them were greater than 0.8 [42], which indicated that the scale had good content validity, with clear and easy-to-understand questions and appropriate entries. We used EFA to measure the structural validity of the scale, which reflects the degree of integration of the scale with the theoretical or conceptual framework on which it is based [48]. The EFA results of the Chinese version of the OHLD scale showed that a total of 3 common factors were extracted, and the cumulative variance contribution rate was 79.794%, indicating that this item had a strong explanatory power for the oral health literacy of diabetic patients. The results of the Chinese version of the OHLD scale showed that all the fitted indicators met the judgment criteria, indicating that the Chinese version of the OHLD scale had a better overall fitting effect. Meanwhile, the CFA results showed that the fitting indices of this scale met or exceeded the fitting indices of the original report. We believe that the Chinese version of the OHLD scale has appropriate validity.

### Limitation

However, this study also has some limitations. Due to conditions and time constraints, the subjects selected for this study were only the inpatients of the endocrinology department of two tertiary hospitals in Liaoning Province, and further investigations are still needed to determine whether the scale is applicable to other provinces at a later stage. Future studies may expand the collection to the whole country and take into account large, medium, and small cities as well as rural hospitals. We have not yet explored the factors affecting the oral health behaviors of diabetic patients. Therefore, this will be the focus of our future work and is very important for our next steps.

## Conclusion

The English version of the OHLD scale has been successfully translated and adapted to Chinese culture, and its psychometric properties have been validated among diabetic patients. In addition, factor analyses showed that the Chinese version of the OHLD scale was dimensionally consistent with the original scale and was reliable and valid. In the context of the Healthy China strategy, the scale can effectively assess oral health literacy among diabetic patients, create educational initiatives and research interventions, and improve the quality of care services.

## Data Availability

The datasets used and/or analysed during the current study available from the corresponding author on reasonable request.
